# Cross-cultural nuances in sarcasm comprehension: a comparative study of Chinese and American perspectives

**DOI:** 10.3389/fpsyg.2024.1349002

**Published:** 2024-02-20

**Authors:** Yiran Du, Huimin He, Zihan Chu

**Affiliations:** ^1^Harvard Graduate School of Education, Harvard University, Cambridge, MA, United States; ^2^English Language Centre, School of Languages, Xi’an Jiaotong-Liverpool University, Suzhou, China; ^3^College of Life Sciences, Henan Normal University, Xinxiang, Henan, China

**Keywords:** sarcasm, sarcasm comprehension, irony, figurative language, national culture

## Abstract

It is evident that sarcasm can be interpreted differently due to various factors, However, rare research was conducted to investigate the influence of national culture on sarcasm comprehension despite its valuable theoretical implication. This study used an online rating task to explore how national culture impacts the comprehension of sarcasm, focusing on the differences between Chinese and American cultural values (i.e., power distance, uncertainty avoidance, collectivism, long-term orientation, and masculinity) and their influence on comprehending sarcastic praise and criticism. The study showed that Chinese participants tend to understand sarcasm less than Americans. It also found that Power Distance is linked to better sarcasm comprehension in both cultures, while Uncertainty Avoidance has a negative effect on it, especially in Chinese participants. Collectivism is also associated with improved sarcasm comprehension, especially in Chinese participants. However, Masculinity and Long-Term Orientation do not seem to have a significant impact on sarcasm comprehension, regardless of nationality or the type of comment (praise or criticism). Overall, the study reveals nuanced differences in how cultural values shape the comprehension of sarcasm in Chinese and American contexts, underscoring the complex interplay between culture and communication.

## 1 Introduction

### 1.1 Sarcasm comprehension

Irony and sarcasm are types of figurative language that are generally used to convey a message that is the opposite of what is explicitly stated (Garcia et al., 2022). Sarcasm is a particular type of irony that is employed when the subject of the remark is an individual (Kreuz and Glucksberg, 1989) and is the focus of the current study. There are two types of sarcasm: sarcastic criticism and sarcastic praise (Bruntsch and Ruch, 2017). Sarcastic criticism is employed to critique someone (e.g., saying, “What a punctual person you are!” to a team member who shows up late for a meeting), but sarcastic praise serves to compliment (e.g., exclaiming, “You’re absolutely hopeless at basketball!” to a buddy who insists they are not good at the sport, but then goes on to win a major tournament). Sarcasm can be indicated through various communicative signals, including contextual, verbal, and paralinguistic/nonverbal cues: contextual cues emphasize the contrast between the utterance and the circumstances, verbal cues are the verbal marker that frequently go along with sarcastic comments such as exaggerated adverbs and adjective, and paralinguistic cues are the nonverbal indicators often linked with sarcasm such as alterations in voice tone and facial expressions (Garcia et al., 2022). Moreover, the meaning of sarcastic statements could vary due to the impacts of individual differences of senders [e.g., age (Brewer et al., 1981); gender (Hoffman and Hurst, 1990); occupation (Pratto and Bargh, 1991; Cui et al., 2023)] and receivers [e.g., age, (Howman and Filik, 2020; Garcia et al., 2022); theory of mind (Blasko and Kazmerski, 2006; Tiv et al., 2023); working memory capacity (Olkoniemi et al., 2016, 2019); personal trait (Mewhort-Buist and Nilsen, 2013)] as well as the sociocultural context (Blasko et al., 2021; Zhu and Filik, 2023). However, relatively few studies explored sarcasm comprehension cross-culturally to data (Banasik-Jemielniak and Kałowski, 2022; Zhu and Filik, 2023), To fill the gap, the current study aims to explore how national culture impact sarcasm comprehension in both Chinese and American groups.

### 1.2 National culture

To describe national culture and the differences of various national cultures, researchers proposed some models. For example, Hofstede (2001) proposed a five-dimensional measure of cultural values: (1) individualism versus collectivism, (2) societal masculinity versus femininity, (3) low versus high power distance, (4) low versus high uncertainty avoidance, and (5) short-versus long-term orientation. To tackle the conceptual issue of uniformly applying national scores to individuals, Yoo et al. (2011) then developed the 26-item CVSCALE based on Hofstede’s original questionnaires and associated derivative works, which is used to measure an individual’s preference for each national cultural dimension (power distance, uncertainty avoidance, collectivism, long-term orientation, and masculinity). Banasik-Jemielniak and Kałowski (2022) argue that when conducting cross-cultural research on sarcasm comprehension, it’s important to incorporate measures of national cultural dimensions or behavioral patterns at an individual level instead of presuming that the wide-ranging differences in national-level scores will automatically reflect the differences between the samples.

Hofstede (1980) characterized individualism by the prioritization of individual rights over obligations, attention to self and immediate kin, emphasis on personal freedom and self-realization, and identity formation through personal achievements. Waterman (1984) described normative individualism as entailing personal accountability and the liberty of decision-making, fulfilling one’s own potential, and honoring others’ rights. Schwartz (1990) described societies with individualistic tendencies as inherently based on agreements, where small core groups and clear-cut social engagements prevail, with particular responsibilities and anticipations aimed at attaining prestige. Instead of viewing individualism and collectivism as mere opposites, a more nuanced perspective suggests they represent distinct worldviews that highlight different concerns (Kagitcibasi, 1987; Kwan et al., 1997). Schwartz (1990) portrays collectivist societies as integrated communities where there are broad, reciprocal roles and expectations shaped by inherent social standings. Within such societies, groups sharing a common destiny, objectives, and beliefs take a central place; the individual is seen as an integral part of the collective, thus prioritizing the group as the primary subject of study (Oyserman et al., 2002). This perspective emphasizes collectivism as an inherently social orientation that favors in-group relationships over out-group ones (Oyserman, 1993).

Masculinity versus Femininity, understood as characteristics of societies rather than individuals, pertains to how different cultures allocate values across genders. This represents a core concern for societies, for which various resolutions may be observed (Hofstede, 2011). The proactive end of the spectrum is often labeled as ‘masculine’, characterized by assertiveness, while the gentle, nurturing end is termed ‘feminine’. In societies considered feminine, both women and men share these nurturing and modest values; conversely, in societies deemed masculine, women may exhibit assertiveness and competitiveness, yet typically to a lesser degree than men, resulting in a distinct divergence between the values of the two genders (Hofstede, 2011).

Power distance is referred to as the extent to which inequalities are accepted in societies (Hofstede, 1980, 2001). This concept is particularly relevant in organizational settings, as power within organizations is inherently distributed unequally (Farh et al., 2007). Power distance affects how much participative decision-making is used, the degree of centralization, and the level of formal hierarchy in organizations (Hofstede, 2001). In cultures with a high power distance, those in power are often viewed as superior, unapproachable, and paternalistic, and they are expected to lead in an autocratic manner (Hofstede, 1980). In such environments, individuals with less power tend to accept their position in the hierarchy, trust their leaders, rely on their judgments (Kirkman et al., 2009), and typically exhibit submissiveness, loyalty, and obedience toward their superiors (Bochner and Hesketh, 1994). Additionally, high power distance is linked to a greater focus on tasks over people, as cultures with high power distance prioritize structured approaches for task completion while maintaining the social distances present in hierarchical relationships (Bochner and Hesketh, 1994).

Uncertainty Avoidance relates to a culture’s comfort with ambiguity, distinguishing between societies that condition their members to feel uneasy or at ease with situations that are unfamiliar, unexpected, or deviate from the norm. Cultures that avoid uncertainty tend to reduce the occurrence of these scenarios through stringent norms, regulations, and laws, discouraging unconventional views and often holding a conviction that there is only one absolute truth which they possess (Hofstede, 2011). Studies indicate that individuals from countries that prefer to avoid uncertainty tend to be more expressive emotionally and driven by internal anxiety. In contrast, cultures that are more accepting of uncertainty are generally more open to diverse viewpoints, favor minimal regulations, and embrace empirical and relativist attitudes in philosophy and religion, allowing for a variety of beliefs to coexist. People in these societies often display a calm and reflective demeanor, with cultural norms that do not necessitate overt emotional expression.

Long-Term Orientation (LTO), as initially conceptualized, contrasted a future-focused perspective with a present or past focus, essentially distinguishing between long-term and short-term views (Hofstede, 1991). However, this conceptualization encountered some complexities, particularly in terms of certain attributes like respect for tradition and learning from past experiences. In the Value Survey Module 94 questionnaire, Hofstede (1994) indicated that respect for tradition aligns with short-term values, whereas persistence aligns with long-term values. This differentiation led to confusion regarding the construct’s application. Fang (2003) documented the evolution of LTO, noting a shift from its original alignment with Confucian values. Hofstede (2001) himself made a significant change in his second edition of “Culture’s Consequences,” no longer using ‘long-term orientation’ and ‘Confucian dynamism’ interchangeably and identifying LTO as the fifth cultural dimension. Subsequently, researchers have reinterpreted LTO as a cultural value emphasizing a holistic view of time, incorporating both the future and the past, rather than focusing solely on immediate or short-term impacts and this redefined LTO includes the importance of planning, valuing traditions, working hard for future gains, and perseverance (Bearden, 2006). This view aligns with Earley’s (1997) conceptualization of LTO as a time-oriented cultural construct.

### 1.3 The impact of national culture on sarcasm comprehension

The comprehension and use of sarcasm could vary across different cultures (Banasik-Jemielniak and Kałowski, 2022). For example, individuals from collectivist societies are generally more inclined to use and seek out implied meanings compared to those from individualistic cultures (Holtgraves, 1997). Individualism has also been linked to the social acceptability of expressing negative emotions, which could potentially lead to more positive interpretations of ironic statements, given their potential use for assertive communication, conveying negative attitudes, or managing hierarchical distance (Fernández et al., 2000). Filippova (2014) demonstrated cultural differences in the understanding of sarcasm between Czech and Canadian participants, which included both children and adults. Canadians found sarcastic praise more humorous than the Czechs did. Conversely, Czechs found sarcastic praise more challenging to comprehend compared to sarcastic criticism. Simpson (2019) demonstrated differences in the understanding of situational irony between North American and UK respondents. While North Americans and UK participants similarly identified situations as ironic, the proportions varied for each individual story. Finally, Zhu and Filik’s (2023) study explored individual variances in the interpretation and usage of sarcasm in the UK and China. Participants evaluated both literal and sarcastic comments in terms of perceived sarcasm, aggression, amusement, and politeness. Regarding interpretation, UK participants perceived sarcasm as more amusing and polite compared to literal criticism. However, for the Chinese participants, sarcasm was seen as more amusing but also more aggressive than literal criticism. Despite the examination of some cross-cultural studies, the influence of cultural variables has been predominantly disregarded (Zhu and Filik's, 2023), notwithstanding considerable theoretical indications of its importance, which underscores the necessity to integrate a cross-cultural perspective in the field of psycholinguistic sarcasm research (Banasik-Jemielniak and Kałowski, 2022).

### 1.4 The present study

In summary, relatively few studies explored sarcasm comprehension cross-culturally so far and there is rare research investigating how cultural dimensions (i.e., power distance, uncertainty avoidance, collectivism, long-term orientation, and masculinity) relate to sarcasm comprehension across western and eastern culture. Hence, the current research aims to explore whether the national culture will impact sarcasm comprehension. Specifically, this study investigated the differences of national culture values and sarcastic praise and criticism comprehension between Chinese and American. It also explored how five cultural dimensions (i.e., power distance, uncertainty avoidance, collectivism, long-term orientation, and masculinity) are associated with sarcastic praise and criticism comprehension in Chinese and American groups.

The present study hypothesizes that Chinese and American participants in this study will show significant differences in certain cultural values (Yoo et al., 2011). Based in previous research on the impact of national culture on sarcasm comprehension (e.g., Simpson, 2019; Zhu and Filik's, 2023), this study predicts that there will be significant difference of sarcasm comprehension between Chinese and American groups. However, due to no main effect of valence reported in previous research (e.g., Garcia et al., 2022), there will not be significance difference between comprehension of sarcastic praise and criticism in both Chinese and American participants. Moreover, in high power distance cultures, where hierarchy and respect for authority are emphasized, individuals might be more cautious and attentive to nuances in communication to avoid misinterpreting messages that could disrupt hierarchical relationships (Hofstede, 1980, 2001). This attentiveness could potentially enhance their ability to comprehend sarcasm. Therefore, this study hypothesize that power distance will be positively associated with sarcasm comprehension. Cultures with high uncertainty avoidance prefer clear, direct communication and are less comfortable with ambiguity (Hofstede, 1980, 2001). This will lead to lower comprehension of sarcasm, which often relies on ambiguity and indirect meanings. Given that people from collectivist cultures tend to prefer and actively search for meanings that are implied or indirect, in contrast to individuals from individualistic societies who are less inclined toward such communication styles (Holtgraves, 1997), collectivism will positively predict sarcasm comprehension score.

## 2 Methods

### 2.1 Participants

In this study, 48 Chinese participants were recruited from Xi’an Jiaotong-Liverpool University. All were enrolled in high-level English for Academic Purposes (EAP) classes and proficient in English, with proficiency levels above C1 according to the Common European Framework of Reference for Languages (CEFR). Among them, 19 were male (39.6%), 26 were female (54.2%), and 3 preferred not to disclose their gender (6.3%). Their ages ranged from 18 to 24 years. Additionally, 48 participants from the United States were gathered for the study through social media advertisements that included a link to an online survey. Of these participants, 18 were male (37.5%), 27 were female (56.3%), and 3 identified as third gender or non-binary (6.3%). The majority were aged between 18 and 24 (43 participants, 89.6%), while 5 reported being 25 to 30 (10.4%). The sample size for this study was determined from prior research that also examined sarcasm comprehension in both younger (*n* = 48) and older adults (*n* = 48) using similar stimuli (Garcia et al., 2022).

### 2.2 Materials and design

48 experimental items from Garcia et al. (2022) were modified and then adopted in this study. Each item represented an interaction between two entities, comprising two sentences. The introductory sentence set the backdrop (e.g., Aimee noticed that Abi’s drawing wasn’t very good), while the succeeding sentence incorporated a comment transmitted from one entity to the other (e.g., She texted Abi to say, “Great drawing.”). Depending on the context provided by the first sentence, the accurate comprehension of the transmitted comment could either be literal or sarcastic, and could be intended as either criticism or praise. Therefore, the research adopted a 2 (literality: literal vs. sarcastic) × 2 (valence: criticism vs. praise) × 2 (nationality: America vs. China) mixed design. Participants were exposed to 4 different versions of the questionnaire, each reflecting a unique combination of literality and valence. Every participant was presented with 48 experimental items—12 items corresponding to each of the 4 conditions—supplemented by 24 filler items. To mitigate potential sequence effects, the presentation order of these items was randomized for each respondent. Each item was succeeded by a question accompanied by a response scale ranging from one to eight (e.g., Will Abi think Aimee disliked her drawing? Very Unlikely 1 2 3 4 5 6 7 8 Very Likely). The scale measured participants’ abilities to comprehend sarcasm correctly. Yoo et al.’s (2011) CVSCALE, a 26-Item Five-Dimensional Scale of Individual Cultural Values was also included in this study. The scale was used to measure sarcasm comprehension where a higher score suggested that the comment was comprehended as more sarcastic. The assessment of the cultural values (i.e., power distance, uncertainty avoidance, collectivism, long-term orientation, and masculinity) utilized 7-point Likert-type scales. For the long-term orientation, the scale ranged from 1, labeled as “very unimportant,” to 7, marked as “very important.” For the other dimensions, the scale spanned from 1, indicating “strongly disagree,” to 7, signifying “strongly agree.” Finally, participants’ nationality, gender, and age were investigated.

### 2.3 Procedure

Participants accessed the study through a URL link hosted by Qualtrics.^1^ Prior to taking part, they were informed that their participation was voluntary, they could withdraw from the study at any time, and that their data would be used for research purposes only and kept confidential. They were then presented with the General Data Protection Regulation and consent form, which required that they indicate their consent before the study would commence. The study began with questions regarding participants’ nationality, gender, and age, after which they were required to complete CVSCALE. Finally, participants were instructed to read the short scenarios and following each scenario answer the questions. Upon completion of the study, participants were debriefed and to thank them for their involvement.

### 2.4 Data analysis

Python (version 3.8.10) was used for quantitative data analysis. Descriptive statistics was calculated for nationality, gender, age, CVSCALE, and sarcasm comprehension score in different conditions. Reliability test was conducted to assess the reliability of scales measuring five cultural values Cronbach’s Alpha, ensuring acceptable reliability. Independent samples t-tests were conducted to identify significant differences across cultural dimensions. A two-way ANOVA was employed to explore the effects of nationality and valence on sarcasm comprehension. Lastly, a multiple linear regression model was utilized to investigate the impact of cultural dimensions and their interactions with nationality and valence on sarcasm comprehension. These methods provided a comprehensive analysis of the cultural differences and their influence on understanding sarcasm.

## 3 Results

### 3.1 Differences of national culture values and sarcasm comprehension between Chinese and American

The reliability of the scales for five cultural values was examined using Cronbach’s Alpha as the indicator (refer to Table 1). The values of Cronbach’s Alpha, calculated from Chinese, American, and pooled samples, were all above 0.700, indicating acceptable reliability. Moreover, the CVSCALE in showed reliable and valid performance across diverse countries (the U.S., South Korea, Brazil, and Poland) and different sample types in Yoo et al.’s (2011) study. Its consistent psychometric properties in various settings and its confirmation in 13 independent studies highlight its widespread applicability and generalizability. Independent samples t-tests were conducted to assess differences across five cultural dimensions. The tests indicated statistically significant differences in three dimensions. For Uncertainty Avoidance, a notable difference was found between Chinese (*M* = 5.34) and American (*M* = 4.93) respondents, *t*(94) = 3.172, *p* = 0.0017 < 0.05. Collectivism also showed significant disparity, with Chinese scoring higher (*M* = 4.25) than Americans (*M* = 3.90), *t*(94) = 2.572, *p* = 0.0106 < 0.05. Long-Term Orientation exhibited a similar trend, with Chinese (*M* = 3.69) outscoring Americans (*M* = 3.41), *t*(94) = 2.463, *p* < 0.05. Moreover, Masculinity showed a marginally significant difference and was higher for Americans (Chinese *M* = 5.22, American *M* = 5.45, *t*(94) = −1.908, *p* = 0.0573). However, there were no significant differences in Power Distance (Chinese *M* = 2.79, American *M* = 2.63, *t*(94) = 1.396, *p* = 0.164). These results indicate significant cultural variations between Chinese and American samples in this study in terms of uncertainty avoidance, collectivism, masculinity, and long-term orientation, while comprehensions of power distance are comparatively similar.

**Table 1:** CVSCALE results in Chinese (*n* = 48) and American groups (*n* = 48).

Scale content and reliability (Cronbach’s Alpha)	Chinese	American
*Power Distance (PD; C = 0.843, A = 0.714, p = 0.750)*	*2.79*	*2.63*
People in higher positions should make most decisions without consulting people in lower positions (PD1)	3.27	2.58
People in higher positions should not ask the opinions of people in lower positions too frequently (PD2)	2.60	3.33
People in higher positions should avoid social interaction with people in lower positions (PD3)	2.04	2.15
People in lower positions should not disagree with decisions by people in higher positions (PD4)	2.81	2.27
People in higher positions should not delegate important tasks to people in lower positions (PD5)	3.23	2.79
*Uncertainty Avoidance (UA; C = 0.897, A = 0.830, p = 0.868)*	*5.34*	*4.93*
It is important to have instructions spelled out in detail so that I always know what I’m expected to do (UA1)	5.08	5.02
It is important to closely follow instructions and procedures (UA2)	5.46	4.50
Rules and regulations are important because they inform me of what is expected of me (UA3)	5.17	4.38
Standardized work procedures are helpful (UA4)	5.35	5.19
Instructions for operations are important (UA5)	5.62	5.54
*Collectivism (CV; C = 0.892, A = 0.779, p = 0.855)*	*4.25*	*3.90*
Individuals should sacrifice self-interest for the group (CV1)	3.79	3.33
Individuals should stick with the group even through difficulties (CV2)	4.96	4.56
Group welfare is more important than individual rewards (CV3)	4.62	3.94
Group success is more important than individual success (CV4)	4.25	4.04
Individuals should only pursue their goals after considering the welfare of the group (CV5)	4.00	3.54
Group loyalty should be encouraged even if individual goals suffer (CV6)	3.90	4.00
*Long-Term Orientation (LTO; C = 0.767, A = 0.707, p = 0.702)*	*3.69*	*3.41*
Careful management of money (Thrift) (LTO1)	2.54	1.60
Going on resolutely in spite of opposition (Persistence) (LTO2)	3.23	2.50
Personal steadiness and stability (LTO3)	3.31	2.52
Long-term planning (LTO4)	3.29	3.56
Giving up today’s fun for success in the future (LTO5)	5.00	5.50
Working hard for success in the future (LTO6)	4.77	4.79
*Masculinity (ML; C = 0.830, A = 0.719, p = 0.780)*	*5.22*	*5.45*
It is more important for men to have a professional career than it is for women (ML1)	5.10	5.79
Men usually solve problems with logical analysis; women usually solve problems with intuition (ML2)	5.25	5.60
Solving difficult problems usually requires an active, forcible approach, which is typical of men (ML3)	4.71	4.60
There are some jobs that a man can always do better than a woman (ML4)	5.81	5.81

C, Chinese; A, American; and P, the pooled sample.

**Figure 1:**
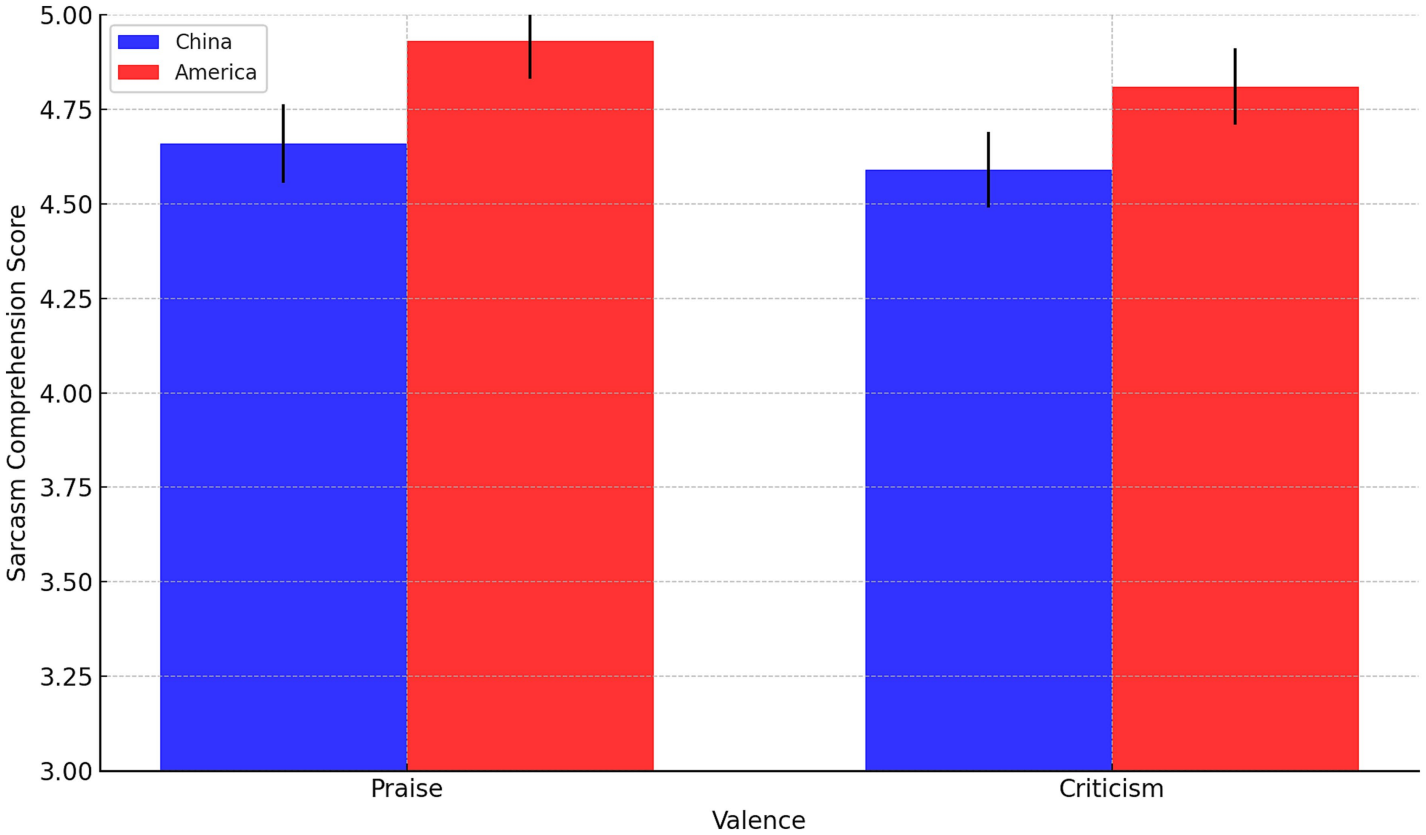
Mean sarcasm comprehension scores by nationality and valence, with error bars representing ±1 standard error of the mean.

A two-way ANOVA was conducted to explore the influences of nationality (China vs. America) and valence (praise vs. criticism) on the comprehension of sarcasm (see Figure 1). The factors nationality (China = −1, America = 1) and valence (Praise = −1; Criticism = 1) were coded using sum coding. The results indicated a significant main effect of nationality on the comprehension of sarcasm, *F*(1, 94) = 6.03, *p* = 0.014 < 0.05. This suggests that there are statistically significant differences in the comprehension of sarcasm between Chinese and American participants. Specifically, Chinese participants (*M* = 4.66, *SE* = 0.104 for praise; *M* = 4.59, *SE* = 0.100 for criticism) tended to rate comments as less sarcastic compared to American participants (*M* = 4.93, *SE* = 0.099 for praise; *M* = 4.81, *SE* = 0.101 for criticism). In contrast, the main effect of valence on sarcasm comprehension was not statistically significant, *F*(1, 94) = 0.92, *p* = 0.336. Additionally, the interaction effect between nationality and valence on sarcasm comprehension was also found to be non-significant, *F*(1, 94) = 0.04, *p* = 0.837. These findings imply that while the nationality of the participant plays a role in how sarcasm is perceived, the nature of the sarcastic comment (whether it is in the form of praise or criticism) does not significantly influence this comprehension, nor does the interaction between nationality and valence.

### 3.2 The impact of five cultural dimensions on sarcasm comprehension in Chinese and American groups

A multiple linear regression model, *Sarcasm Comprehension* = *β_0_* + *β_1_* × *Power Distance* + *β_2_* × *Uncertainty Avoidance* + *β_3_* × *Collectivism* + *β_4_* × *Long-Term Orientation* + *β_5_* × *Masculinity* + *β_6_* × (*Nationality* × *Power Distance*) + *β_7_* × (*Nationality* × *Uncertainty Avoidance*) + *β_8_* × (*Nationality* × *Collectivism*) + *β_9_* × (*Nationality* × *Long-Term Orientation*) + *β_10_* × (*Nationality* × *Masculinity*) + *β_11_* × (*Valence* × *Power Distance*) + *β_12_* × (*Valence* × *Uncertainty Avoidance*) + *β_13_* × (*Valence* × *Collectivism*) + *β_14_* × (*Valence* × *Long-Term Orientation*) + *β_15_* × (*Valence* × *Masculinity*) + *ε*, was built to explore the impact of five cultural dimensions and their interaction with nationality (China vs. America) and Valence (Praise vs. Criticism) on sarcasm comprehension. The factor nationality and valence were coded using indicator coding with Americans and praise as the reference groups, respectively.

Several notable findings were observed (see Table 2). The analysis revealed that Power Distance positively correlates with sarcasm comprehension (*b* = 0.28, *SE* = 0.11, 95% CI [0.14, 0.35], *t* = 2.74, *p* < 0.05). This suggests that individuals in societies with more pronounced hierarchical structures tend to have a better understanding of sarcasm. However, the interaction (China × Power Distance) did not show significant differences, indicating that the effect of Power Distance on sarcasm comprehension is consistent across both Chinese and American contexts.

**Table 2:** Results of the multiple linear regression model and the parameters.

Parameter	*b*	*SE*	95% CI Lower	95% CI Upper	*t*
(Intercept)	4.56	0.78	3.04	6.09	5.87***
Power distance	0.28	0.11	0.14	0.35	2.74*
China × Power distance	−0.09	0.12	−0.32	0.15	−0.72
Criticism × Power distance	0.05	0.11	−0.16	0.26	0.47
Uncertainty avoidance	−0.35	0.10	−0.54	−0.16	−3.65***
China × Uncertainty avoidance	−0.55	0.11	−0.78	−0.33	−4.88***
Criticism × Uncertainty avoidance	−0.01	0.11	−0.22	0.19	−0.13
Collectivism	0.36	0.10	0.15	0.55	3.66***
China × Collectivism	0.43	0.11	0.20	0.65	3.78***
Criticism × Collectivism	−0.22	0.11	−0.43	−0.01	−0.06
Long-term orientation	−0.37	0.13	−0.64	−0.11	−0.78
China × Long-term orientation	0.44	0.14	0.16	0.72	0.05
Criticism × Long-term orientation	0.28	0.13	0.02	0.54	0.15
Masculinity	0.02	0.10	−0.17	0.22	0.24
China × Masculinity	−0.12	0.12	−0.36	0.11	−1.01
Criticism × Masculinity	0.13	0.11	−0.08	0.35	1.21

*p < 0.05, **p < 0.01, ***p < 0.001. CI, Confidence Interval.

Uncertainty Avoidance demonstrated a negative relationship with sarcasm comprehension (*b* = −0.35, *SE* = 0.10, 95% CI [−0.54, −0.16], *t* = −3.65, *p* < 0.001). This finding implies that in societies where there is a high need to avoid uncertainty, individuals may have a reduced capacity to understand sarcasm. The interaction of Uncertainty Avoidance with nationality (China × Uncertainty Avoidance: *b* = −0.55, *SE* = 0.11, 95% CI [−0.78, −0.33], *t* = −4.88, *p* < 0.001) was significantly negative. This indicates a more pronounced negative effect in the Chinese context compared to the American context, suggesting that cultural factors in China may exacerbate the challenges in comprehending sarcasm related to uncertainty avoidance. The interaction with valence (Criticism × Uncertainty Avoidance) was not significant, showing that the effect of Uncertainty Avoidance on sarcasm comprehension does not vary substantially between praise and criticism.

The model also found a positive correlation between Collectivism and sarcasm comprehension (*b* = 0.36, *SE* = 0.10, CI [0.15, 0.55], *t* = 3.66, *p* < 0.001), hinting that collectivist cultures might have an enhanced understanding of sarcasm. The significant interaction with nationality (China × Collectivism: *b* = 0.43, *SE* = 0.11, 95% CI [0.2, 0.65], *t* = 3.78, *p* < 0.001) underscores a stronger effect in the Chinese context compared to American context. However, the interaction with valence (Criticism × Collectivism) did not show a significant effect, suggesting that the comprehension of sarcasm is not heavily influenced by whether the sarcasm is framed as praise or criticism.

The study found that neither Masculinity nor Long-Term Orientation had a significant impact on sarcasm comprehension. This lack of significant findings indicates that the traits typically associated with these cultural dimensions – such as assertiveness, competitiveness, persistence, and thrift – do not have a notable influence on the ability to comprehend sarcasm. Furthermore, their interactions with nationality (China vs. America) and valence (Criticism vs. Praise) were also not significant, suggesting a uniformity in the effect (or lack thereof) of these cultural dimensions on sarcasm comprehension across different national and valence contexts.

## 4 Discussion

The study examines how national culture, particularly in Chinese and American contexts, affects the understanding of sarcastic praise and criticism, focusing on the influence of five cultural dimensions: power distance, uncertainty avoidance, collectivism, long-term orientation, and masculinity. The study revealed significant differences in cultural values and sarcasm comprehension between Chinese and American participants. Three cultural dimensions – Uncertainty Avoidance, Collectivism, and Long-Term Orientation – showed notable differences between the two groups, with Chinese scoring higher in all three. In terms of sarcasm comprehension, Chinese participants were found to perceive comments as less sarcastic compared to Americans. The research also indicated that Power Distance positively correlates with sarcasm comprehension across both cultures, while Uncertainty Avoidance negatively impacts it, especially in the Chinese context. Collectivism positively correlates with sarcasm comprehension, particularly in China. However, Masculinity and Long-Term Orientation did not significantly impact sarcasm comprehension, nor did their interactions with nationality or valence (praise vs. criticism).

In this study, the decision to utilize Yoo et al.’s (2011) CVSCALE to investigate the cultural values of Chinese and American samples, rather than presuming broad cultural differences between these nationalities, represents a methodologically robust approach that aligns with current trends in cross-cultural research. This approach, as advocated by Banasik-Jemielniak and Kałowski (2022), emphasizes the importance of assessing individual cultural dimensions over general national stereotypes. By focusing on individual-level cultural values, this study offers a more nuanced and precise understanding of how specific cultural dimensions influence sarcasm comprehension. This method acknowledges the diversity and variability within cultural groups, avoiding the pitfalls of overgeneralization. The findings, showing notable differences in certain cultural dimensions between Chinese and American participants, underscore the complexity and individuality of cultural influences on communication styles. This approach not only enhances the accuracy of cross-cultural comparisons in sarcasm comprehension but also contributes valuable insights to the literature on the intricate interplay between culture and communication. The study’s revelation that Chinese participants generally perceive comments as less sarcastic compared to their American counterparts aligns with the findings of Simpson (2019), who also highlighted the significant role of national culture in the interpretation of sarcasm. Zhu and Filik’s (2023) research further supports this notion, emphasizing that cultural background plays a critical role in how sarcasm is understood, thereby underscoring the importance of considering cultural context in studies of communication styles.

The study’s hypothesis regarding the positive correlation between power distance and sarcasm comprehension finds its roots in Hofstede’s (1991) cultural dimensions theory. In cultures with high power distance, where respect for authority and hierarchy is emphasized, individuals are likely more attuned to nuances in communication to maintain harmonious hierarchical relationships. This heightened attentiveness could potentially enhance their ability to detect and interpret sarcastic comments, a perspective that adds a new dimension to our understanding of the interplay between cultural values and communication styles. Conversely, the finding that uncertainty avoidance negatively impacts sarcasm comprehension, particularly in the Chinese context, resonates with Hofstede’s (1980, 2011) assertions. Cultures characterized by high uncertainty avoidance tend to favor clear and direct communication, finding ambiguity and indirectness less comfortable. This preference may explain the lower sarcasm comprehension among Chinese participants, as sarcasm often relies on subtle and indirect cues, which can be challenging for individuals from such cultures to decipher. Interestingly, the study suggests that collectivism positively correlates with sarcasm comprehension in China, supporting Holtgraves’ (1997) view that collectivist cultures, which value implied and indirect meanings, might be better equipped to understand sarcasm. This finding contrasts with individualistic cultures, where a more straightforward communication style is prevalent, and sarcasm might be less effectively employed or understood. The lack of significant impact of masculinity and long-term orientation on sarcasm comprehension adds another layer to the conversation. It suggests that these particular cultural dimensions might not be as influential in shaping how sarcasm is interpreted, contrasting with other dimensions. Moreover, the study’s observation that there is no significant difference in the comprehension of sarcastic praise versus criticism between Chinese and American participants offers a notable insight. This aligns with Garcia et al. (2022), indicating that the nature of the sarcastic comment (praise or criticism) might not be as critical in understanding sarcasm as the cultural backdrop against which it is delivered.

The current study, focusing on sarcasm comprehension among Chinese and American young adults aged 18–24, presents a solid foundation yet is limited in its scope and methodology. Its concentration on a specific age group and two cultural backgrounds limits the generalizability of its findings to other populations. Moreover, the measure of sarcasm used is simplistic, primarily emphasizing written forms (Garcia et al., 2022), which fails to capture the complexity and multimodal nature of real-life sarcasm. For future research, it is advisable to broaden the demographic and cultural scope, include a more nuanced and diverse set of sarcasm measures encompassing auditory and visual cues, and consider experimental and qualitative methodologies. Specific experimental methodologies like conducting controlled experiments with diverse cultural participants exposed to standardized sarcastic statements would be insightful. Qualitative methods, such as in-depth interviews exploring personal sarcasm experiences, are also recommended. These approaches could clarify the causal relationships between cultural values and sarcasm comprehension. The current study did not employ these methods due to its preliminary nature and resource constraints, focusing instead on broader cultural comparisons. Such approaches would not only enhance the understanding of sarcasm’s role across cultures and ages but also potentially clarify the causal relationships between cultural values and sarcasm comprehension, an aspect that remains underdeveloped in the current study.

Despite the limitations, this study significantly contributes to the understanding of how sarcasm is perceived across different cultures, specifically between Chinese and American individuals. It addresses a notable gap in previous research, which has largely focused on the influence of individual differences in sarcasm comprehension within Western adult populations, and has not adequately considered the impact of national cultural factors (Banasik-Jemielniak and Kałowski, 2022; Zhu and Filik, 2023). By focusing on the interplay between cultural values and sarcasm comprehension, the study provides valuable insights into how national culture impacts communication styles and the interpretation of nonliteral language. This understanding is important for cross-cultural communication, as it highlights the need to consider cultural backgrounds in interpreting verbal interactions. The study underscores the complex relationship between culture and communication, especially in the use and understanding of sarcasm. It highlights the importance of considering both socio-cultural variables and individual characteristics in future research to deepen our understanding of verbal irony across national cultures.

In conclusion, the study offers a groundbreaking contribution to the field of psycholinguistics and cross-cultural communication. Its exploration into how sarcasm is comprehended differently in Chinese and American cultures underscores the profound impact of cultural values on the interpretation of nonliteral language. This study not only fills a significant gap in existing research, which has predominantly focused on Western populations, but also introduces a vital cross-cultural perspective in understanding sarcasm. The findings reveal that cultural dimensions such as power distance, collectivism, uncertainty avoidance, and others play a crucial role in shaping how individuals from different cultures perceive sarcastic comments. This nuanced understanding is pivotal for effective communication across cultures, particularly in our increasingly globalized world where interactions among people from diverse cultural backgrounds are commonplace. Furthermore, the proposed theoretical framework integrating both national cultural and individual psychological factors sets a new direction for future research in this area. It emphasizes the necessity of considering a broader range of variables to fully grasp the complexities of sarcasm use and comprehension across cultures. Overall, this study not only advances our knowledge in the field of psycholinguistics but also has practical implications for enhancing cross-cultural communication. It highlights the importance of cultural awareness and sensitivity in interpreting and using sarcasm, a common yet complex form of verbal expression. As such, it provides valuable insights for educators, communicators, and professionals who operate in multicultural environments, aiding in the development of more effective and nuanced communication strategies.

## Data availability statement

The raw data supporting the conclusions of this article will be made available by the authors, without undue reservation.

## Ethics statement

The studies involving humans were approved by Xi’an Jiaotong-Liverpool University. The studies were conducted in accordance with the local legislation and institutional requirements. The participants provided their written informed consent to participate in this study.

## Author contributions

YD: Conceptualization, Data curation, Investigation, Writing – original draft, Writing – review & editing. HH: Conceptualization, Investigation, Project administration, Resources, Writing – review & editing. ZC: Writing – review & editing.

## Footnotes

^1^ https://www.qualtrics.com
